# Effects of Yttrium Doping on Erbium-Based Hydroxyapatites: Theoretical and Experimental Study

**DOI:** 10.3390/ma15207211

**Published:** 2022-10-16

**Authors:** Lana Omar Ahmed, Niyazi Bulut, Hanifi Kebiroglu, Mohammad Alkhedher, Tankut Ates, Suleyman Koytepe, Burhan Ates, Omer Kaygili, ElSayed M. Tag El Din

**Affiliations:** 1 Department of Physics, Faculty of Science and Health, Koya University, Koya KOY45, Kurdistan Region—F.R., Iraq; 2 Department of Physics, Faculty of Science, Firat University, Elazig 23119, Turkey; 3 Mechanical and Industrial Engineering Department, Abu Dhabi University, Abu Dhabi 111188, United Arab Emirates; 4 Department of Engineering Basic Sciences, Faculty of Engineering and Natural Sciences, Malatya Turgut Özal University, Malatya 44210, Turkey; 5 Department of Chemistry, Faculty of Arts & Science, Inonu University, Malatya 44280, Turkey; 6 Electrical Engineering Department, Faculty of Engineering & Technology, Future University in Egypt, New Cairo 11835, Egypt

**Keywords:** hydroxyapatite, X-ray diffraction, bandgap, spectroscopic analysis, cell viability

## Abstract

This is the first investigation of yttrium (Y) and erbium (Er) co-doped hydroxyapatite (HAp) structures, conducted using theoretical and experimental procedures. By using a wet chemical method, the materials were synthesized by varying the concentration of Y amounts of 0.13, 0.26, 0.39, 0.52, 0.65, and 0.78 at.% every virtual 10 atoms of calcium, whereas Er was kept fixed at 0.39 at.%. Spectroscopic, thermal, and in vitro biocompatibility testing were performed on the generated samples. Theoretical calculations were carried out to compute the energy bandgap, density of states, and linear absorption coefficient. The effects of Y concentration on thermal, morphological, and structural parameters were investigated in detail. Raman and Infrared (FTIR) spectroscopies confirmed the formation of the HAp structure in the samples. Theoretical investigations indicated that the increasing amount of Y increased the density from 3.1724 g cm^−3^ to 3.1824 g cm^−3^ and decreased the bandgap energy from 4.196 eV to 4.156 eV, except for the sample containing 0.39 at. % of the dopant, which exhibited a decrease in the bandgap. The values of linear absorption appeared reduced with an increase in photon energy. The samples exhibited cell viability higher than 110%, which revealed excellent biocompatibility for biological applications of the prepared samples.

## 1. Introduction

Calcium orthophosphates are widely used biomaterials due to their potential for dealing with bone regeneration [1]. One of the most widely utilized forms of these calcium orthophosphates is hydroxyapatite (HAp), which is a promising ceramic material in the form of Ca_10_(PO_4_)_2_(OH)_2_ [2]. HAp is bioactive, biocompatible, and osteoconductive material that is abundantly found in mammalian hard tissues (i.e., bones and teeth). Because of its excellent biological characteristics, HAp has also been employed in a variety of medical applications [3,4,5]. HAp can be synthesized using a variety of methods, including sol–gel technique, solid-state processes, combustion method, microemulsion, spray pyrolysis, mechanochemical route, microwave, and wet chemical route [6,7]. In comparison to the other strategies, the wet chemical approach is a simple and cost-effective way of preparing HAp, which produces good purity samples at low reaction temperatures [8]. HAp is also well-known for the possibility of many anionic and, most of all, cationic substitutions in its structure; considering the potential of the doping process to tailor the properties of host materials, several studies attempted to enhance the properties of HAp [9,10,11,12,13].

Because of their luminescent property, rare-earth elements (REE) can be employed as dopants for biological fluorescence in HAp for several implications [14]. Yttrium has been a widely used REE to enhance the mechanical strength of ceramics [15,16,17]. Y-doped calcium oxyhydroxyapatite has been investigated for use as a humidity sensor due to its greater hydrophilic nature [18]. Furthermore, as compared to pure HAp, Y-doped HAp exhibits higher electrical conductivity [18]. Y-doped HAp is a great candidate for orthopedic implants due to its good electrical conductivity and hydrophilicity [16,18,19,20,21]. Moreover, Y-doped HAp has a high level of stability in a variety of environments and appears to enhance osteoblast adhesion [19,20,22,23]. In addition, the human body contains erbium, with a high concentration in the ribs and a smaller level in the kidneys and liver [24]. According to the researchers, Er-doped HAps could be used in biophotonic applications [25]. Er has also been proposed as a suitable dopant for the HAp structure because of its light emission spectra and good biocompatibility [26].

There have been studies on individual doping of either Y or Er in HAps, but the effects of co-doping of these elements in the host have not yet been carried out. Considering the prospects of the co-doping process to tailor the properties of hosts and applications of HAp, this study was carried out to investigate the effects of Y/Er co-additions using experimental and theoretical methods. By using a wet chemical process, six different concentrations of Y/Er co-doped HAp were synthesized with various concentrations of Y with a fixed Er content in the matrix. The synthesized samples were examined experimentally using X-ray diffraction (XRD), FTIR and Raman spectroscopy, scanning electron microscopy (SEM), and thermal analysis methods. In parallel to the experimental investigations, the calculations of the density of states (DOS) and band structure of Y/Er co-doped HAps were carried out by employing density functional theory (DFT). In addition, the linear absorption coefficient was theoretically calculated for each system to obtain more insights into the studied materials. The studied samples based on detailed experimental and theoretical studies, including biocompatibility measurements, revealed the potential of the materials for biological applications.

## 2. Materials and Methods

### 2.1. Synthesis and Characterization

Wet chemical synthesis was used to efficiently produce Er-based HAp samples with a fixed Er concentration of 0.39 at. % co-doped with Y at amounts 0.13, 0.26, 0.39, 0.52, 0.65, and 0.78 at. %. Distilled water was used as a solvent for the as-used chemicals of calcium nitrate tetrahydrate (Ca(NO_3_)_2_·4H_2_O, Carlo-Erba), erbium nitrate pentahydrate (Er(NO_3_)_3_·5H_2_O, Sigma-Aldrich, St. Louis, MO, USA), yttrium (III) nitrate hexahydrate (Y(NO_3_)_3_·6H_2_O, Sigma–Aldrich) and di-ammonium hydrogen phosphate ((NH_4_)_2_HPO_4_). A 100 mL solution of Ca(NO_3_)_2_·4H_2_O, Er(NO_3_)_3_·5H_2_O and Y(NO_3_)_3_·6H_2_O was prepared in a flask and poured into a beaker. A 100 mL solution of (NH_4_)_2_HPO_4_ was prepared and then poured drop by drop into the initial solution, resulting in the development of a milky solution. The details on the as-used amounts of the aforementioned chemicals for each sample in mole and atomic percentages are given in more detail in Table 1. With including ammonia solution (Sigma-Aldrich), the pH of the solution was adjusted to 10.0, and at the end, the solution was mixed at 65 °C for 75 min. In order to dry the samples, the as-prepared mixture was put in an oven using a temperature of 120 °C for 22 h. The dry powders were calcined for 2.5 h in an electric furnace with a temperature of 900 °C.

A Rigaku Rad B-DMAX II diffractometer was used to obtain XRD data using Cu-Kα radiation at 40 kV and 30 mA in the 2*θ* range from 20° to 60° with a step size of 0.02°. FTIR spectroscopy (PerkinElmer spectrum one spectrophotometer, Waltham, MA, USA) was used in the wavenumber range of 400–4000 cm^−1^ with a step size of 4 cm^−1^ by using the KBr method. The Raman spectra were obtained using Renishaw’s confocal microscope (Wotton-under-Edge, UK) operated via a 532 nm diode laser and maximal power of 10 mW. A Leo Evo-40xVP scanning electron microscope (SEM) was used to study the morphology of the samples coated with gold as a conductive layer. Shimadzu DTA 50 and TGA 50 (Kyoto, Japan) were used to perform DTA and TGA ranging from room temperature to 900 °C at the heating rate of 10 °C min^−1^.

The theoretical analyses were carried out utilizing the CASTEP program to perform quantum calculations of band structure and DOS.

### 2.2. In Vitro Biocompatibility Assay

Under sterile conditions, ceramic powders were weighed separately at 0.1 g each and placed in sterile tubes at a volume of 2 mL. The materials were then given 1 mL of DMEM and placed in the incubator for 72 h. High glucose Dulbecco’s Modified Eagle Medium (DMEM) was used to culture L929 (Mouse Fibroblast) cells in a flask at 37 degrees 5 percent CO_2_. A total of 10,000 L929 cells were put into each well of the 96-well plate. In the CO_2_ incubator, it was supposed to stick and spread until the next day. Twenty-four hours later, the plate was removed and destroyed. Instead, the media that had been in contact with the compounds for 72 h was thoroughly filtered and added to the wells.

For 24 h, cells were cultured in material-suspended media in an incubator. MTT dye was diluted to a concentration of 5 mg/mL in PBS (phosphate buffer, pH 7.4). At the end of 24 h, the soaked medium in the well plate was removed and discarded. Instead, each well was filled with 90 μL of DMEM. MTT (3-(4,5-dimethylthiazol-2-yl)-2,5-diphenyltetrazolium bromide) (3-(4,5-dimethylthiazol-2-yl)-2,5-diphenyltetrazolium bromide) (3-(4,5-dimethylthiazol-2-yl)-2. Each well received 100 mL of dimethyl sulfoxide (DMSO). Immediately after loading DMSO, well plates were examined at 540 nm wavelengths. No animals were harmed during the studies, and in vitro biocompatibility study was performed that did not require ethics committee approval. The statistical analyses for in vitro biocompatibility studies were performed with a GraphPad Prism 8 program “one-way ANOVA” test, and *p* < 0.05 was considered significant in the results. Data were shown as arithmetic mean ± standard deviation.

## 3. Results and Discussion

### 3.1. Experimental Results

The synthesized samples were thoroughly characterized using the mentioned techniques for the study of morphological, structural, thermal stability, and biocompatibility. The measurements, analysis, and discussion of the findings are described in the following. 

#### 3.1.1. XRD Analysis

**Figure 1:**
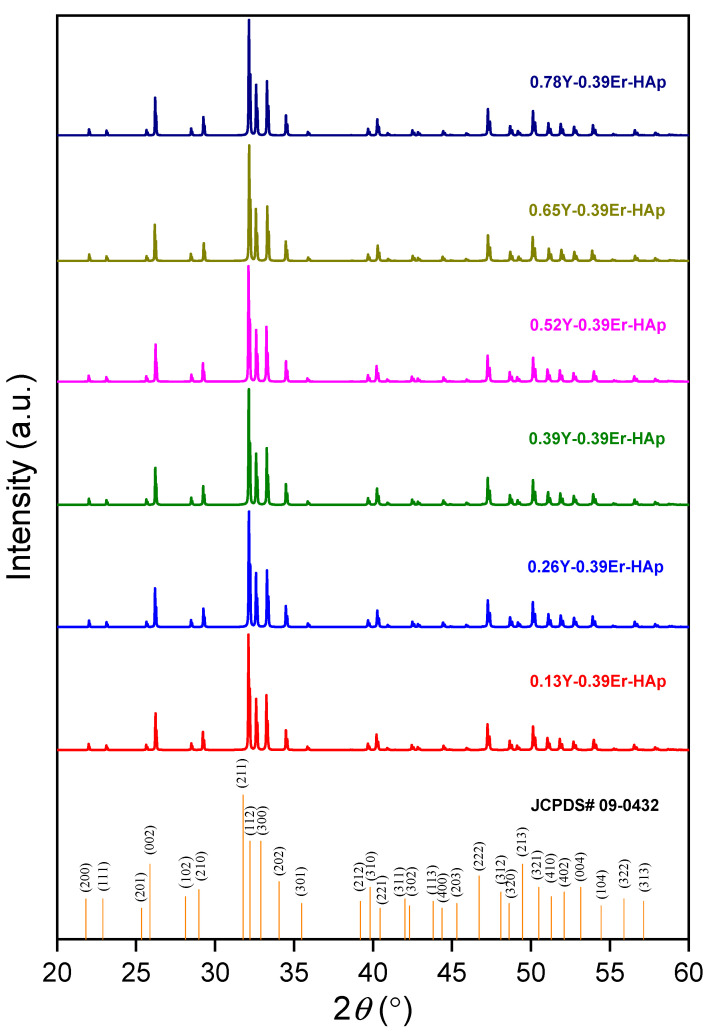
XRD patterns of the samples.

Figure 1 depicts the XRD results of the prepared samples. The analyzed data of the samples were compared with the card for pure HAp (JCPDS no: 09-0432). The XRD plots revealed that HAp appeared in the form of a single phase for all of the samples, without the formation of any possible secondary phase. The occurrence of Er_2_O_3_ and/or Y_2_O_3_-related phases is not observed, probably due to the low total REE amount (1.17at.%). In addition, the XRD data obtained for the samples after the addition of Y caused some variations in the intensity. The impact of ionic doping on the material’s structure leads to bringing changes in peak intensity, as was observed and reported previously [27]. The Scherrer relation given in Equation (1) was used to calculate the crystallite sizes (*D_S_*) [28].

(1) $$D_{s}=\frac{0.9\lambda }{\beta cos\theta }$$

**Figure 2:**
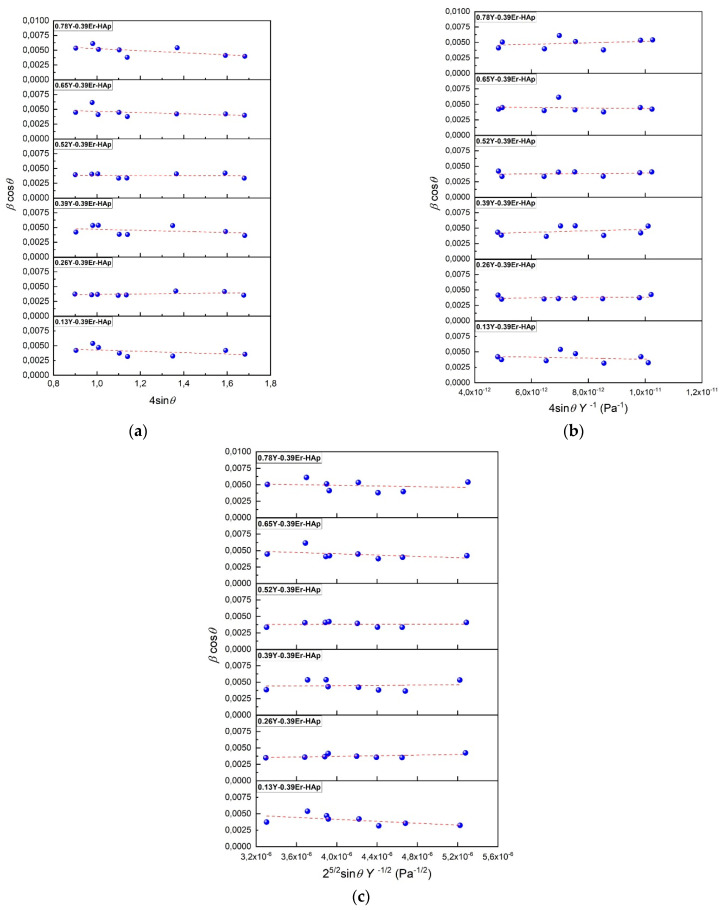
(**a**) The *βcosθ* vs. 4 sin*θ*, (**b**) *βcosθ* vs. 4 sin*θ E*^−1^, and (**c**) *βcosθ* vs. 2^5/2^ sin*θ E*^−1/2^ plots of the Er-based HAps doped with Y.

Further, Williamson–Hall (*D_WH_*) plot was used to evaluate strain as Equation (2) [29].

(2) $$\beta cos\theta =\frac{0.9\lambda }{D_{WH}}+4\varepsilon sin\theta$$

where *β* refers to the full width at half maximum, *λ* is the incoming X-ray source wavelength, theta is the diffraction angle, and *ε* is a lattice strain. The comparison values of the *β*cos*θ* versus 4 sin*θ* for all synthesized samples are shown in Figure 2a. The *D_WH_* and *ε* values can be expressed by using the relationship *ε = σ/E*, where *E* and *σ* denote Young’s modulus and stress, respectively [30].

(3) $$\beta \cos\theta =\frac{0.9\lambda }{D_{WH}}+\frac{4\sigma \sin\theta }{E}$$

The *σ* values for all samples can be obtained from the slope of the *β*cos*θ* versus 4 sin*θ* *E*^−1^ presented in Figure 2b. In order to compute the *E* values, the following relation was used [31]:

(4) $$E=\frac{{\left[h^{2}+\frac{{\left(h+2k\right)}^{2}}{3}+{\left(\frac{al}{c}\right)}^{2}\right]}^{2}}{7.49\times 10^{-12}{\left(h^{2}+\frac{{\left(h+2k\right)}^{2}}{3}\right)}^{2}+10.9\times 10^{-12}{\left(\frac{al}{c}\right)}^{4}-7.1\times 10^{-12}\left(h^{2}+\frac{{\left(h+2k\right)}^{2}}{3}\right){\left(\frac{al}{c}\right)}^{2}}$$

Equation (2) can be modified by substituting (2*u*/*E*)^1/2^ for *ε* to obtain the anisotropic energy density (*u*),

(5) $$\beta \cos\theta =\frac{0.9\lambda }{D_{WH}}+4\sin\theta {\left(\frac{2u}{E}\right)}^{1/2}$$

The *u* values can obtain from the slope of the *βcosθ* versus 2^5/2^ sin*θ E*^−1/2^ in Figure 2c. The *a*, *c*, and *V* (unit cell volume) values are [32].

(6) $$\frac{1}{d^{2}}=\frac{4}{3}\left(\frac{h^{2}+hk+k^{2}}{a^{2}}\right)+\frac{l^{2}}{c^{2}}$$

(7) $$V=0.866a^{2}c$$

where *d* is the inter-planar distance. The crystallinity percentage (*X_C_%*) was computed using Equation (8) via the intensity of the pit (*V*_112/300_) between (112) and (300) reflections and the intensity (*I*_300_) of (300) reflection [33].

(8) $$X_{C}\%=\left(1-\frac{V_{112/300}}{I_{300}}\right)\times 100$$

Theoretical and experimental parameters of *a*, *c*, and *V* are listed in Table 2. These results reveal that the introduction of the Y atom affects these parameters. The experimentally determined values of percent crystallinity (*Xc%*), crystallite sizes (*D_S_* and *D_WH_*), lattice strain *ε*, stress *σ*, and anisotropic energy density (*u*) are listed in Table 3. The addition of Y appeared to change the crystallinity with a value of *Xc*% ranging from 87.4 to 83.7% for the samples. Since Y^3+^ with a high ionic radius (0.1011 nm) [34] and Er^3+^ with a low ionic radius (0.088 nm) [35] are expected to replace Ca^2+^ with an ionic radius of 0.099 nm [28], changes in the crystallite size are likely. The observed changes in crystallite size might be associated with the progression of lattice strain [36]. Furthermore, the distribution of ions Y^3+^, Er^3+^, and Ca^2+^ in the lattice exhibits a charge imbalance. The distribution of Ca in the matrix may have been changed by the placement of Y and Er atoms to the host sites, which influenced the crystallite size. The doping appeared to change *D_S_* and *D_WH_* values which pointed to causing negative and positive lattice strain *ε* in the samples. The unit cell is under compressive stress for negative lattice strain, whereas it exhibits tensile stress in case of positive lattice strain [37]. The doping caused changes in stress (*σ*) and anisotropic energy density (*u*). Due to their proportionality with strain, tensile and compressive stresses give positive and negative values. Except for the 0.39Y-0.39Er-HAp and 0.52Y-0.39Er-HAp samples, whose *u* values are less than the published value of 12 kJ m^−3^, the entire samples exhibited higher *u* values in comparison to this published value [38].

#### 3.1.2. FTIR and Raman Spectroscopy Results

**Figure 3:**
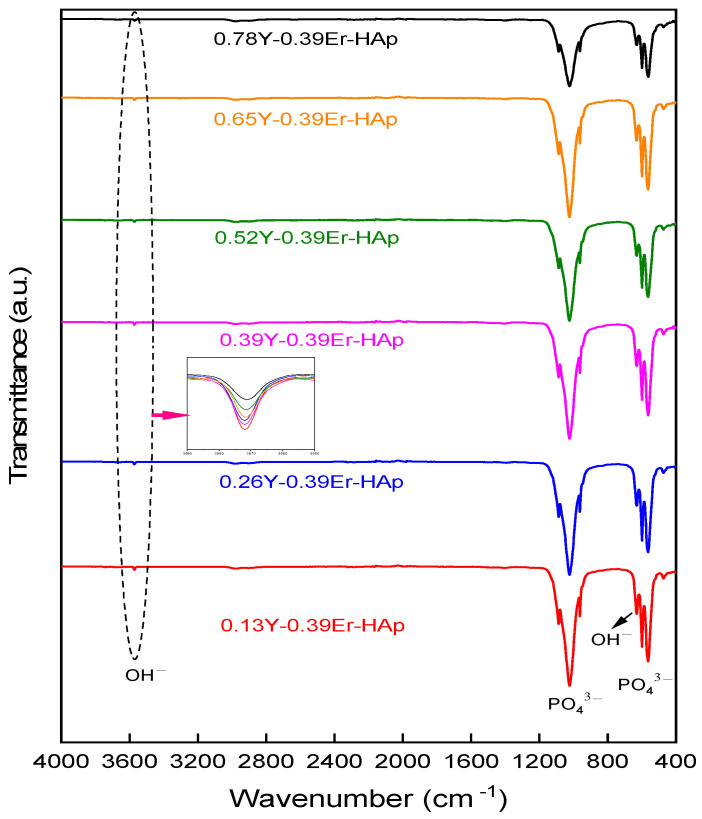
FTIR results of the as-prepared HAps.

FTIR spectra of all the samples are shown in Figure 3, which indicate the presence of phosphate and hydroxyl bands. The vibration modes of the phosphate group were detected at the bands at wavenumber regions of 471 cm^−1^ (symmetric bending, υ_2_), 563 cm^−1^ (asymmetric bending, υ_4_), 597 cm^−1^ (asymmetric bending, υ_4_), 961 cm^−1^ (symmetric stretching, υ_1_), and 1020 cm^−1^ (asymmetric stretching, υ_3_) [39,40]. The vibrational mode of the hydroxyl group is related to the bands at 631 cm^−1^ and 3570 cm^−1^ [3,41]. The presence of the HAp structure was confirmed by all of the as-observed bands in the FTIR spectra for each sample [42].

**Figure 4:**
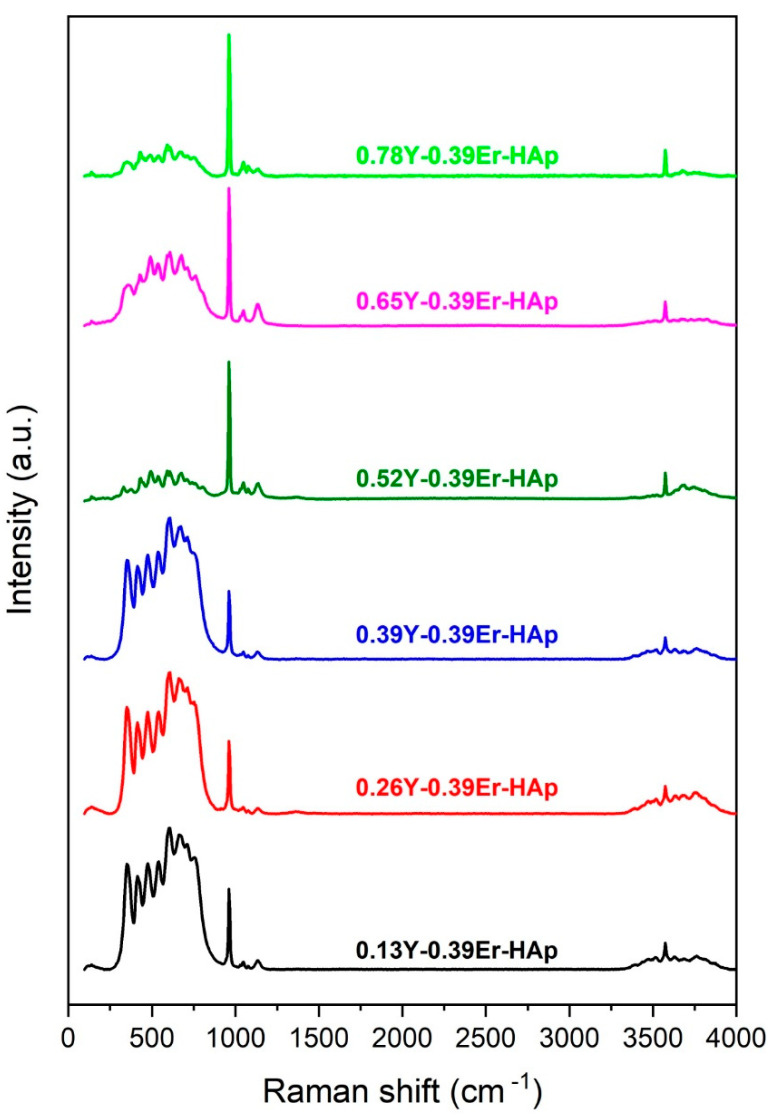
Raman spectra of the as-synthesized HAps.

Figure 4 shows the Raman spectra of the materials. The symmetric and asymmetric O-P-O bending modes of the phosphate group were seen at 426 cm^−1^ and 585 cm^−1^. The symmetric and asymmetric P-O stretching modes were detected at 965 cm^−1^ and 1086 cm^−1^. At 3590 cm^−1^, the band related to the hydroxyl group’s stretching mode was observed.

#### 3.1.3. Morphological Analysis

**Figure 5:**
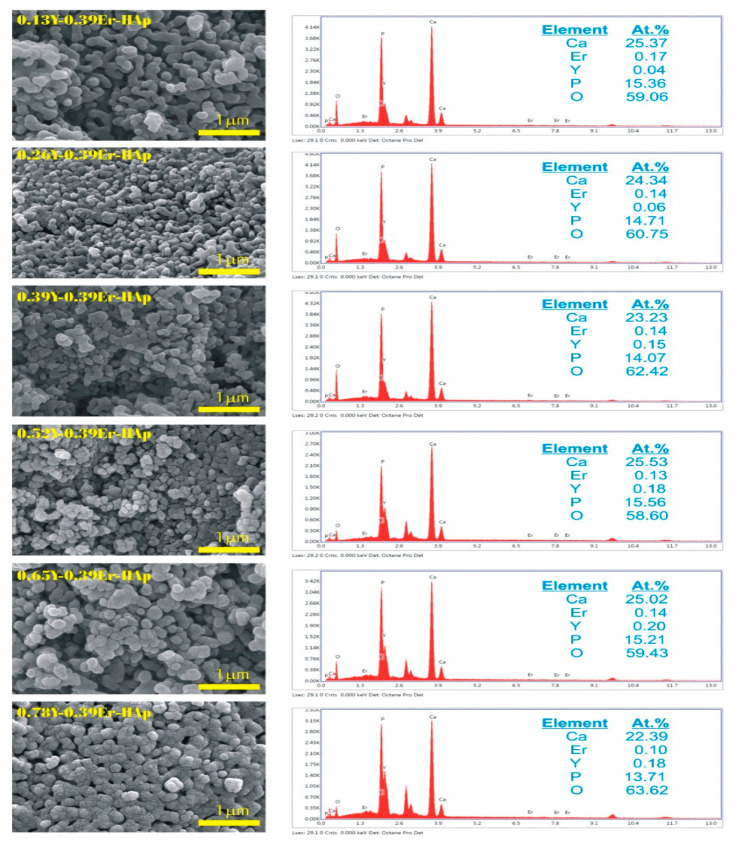
SEM images and EDX analysis.

Figure 5 shows scanning electron microscope (SEM) images and energy-dispersive X-ray (EDX) spectra of as-produced co-doped HAps. The analysis of the morphology of the samples pointed to the presence of sphere-shaped nanoparticles stacked one on top of the other. The EDX results support the introduction of both co-dopants in the HAp structure. Additionally, the influence of these co-dopants is limited in the HAp structure, and this may be due to the deposition of these dopants on the sample surface. Table 4 gives a detailed comparison between experimental and theoretical values of the stoichiometric ratios of Ca/P and (Ca+Er+Y)/P. Both results are in very good agreement with each other. For both molar ratios, the differences between theoretical and experimental values are lower than the value of 1% for all the samples. 

#### 3.1.4. Differential Thermal Analysis (DTA) and Thermogravimetric Analysis (TGA)

**Figure 6:**
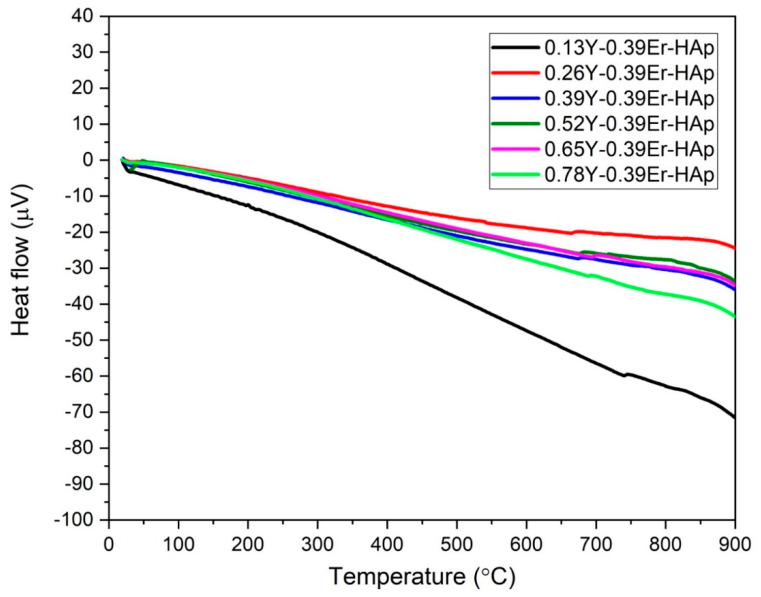
DTA thermograms.

**Figure 7:**
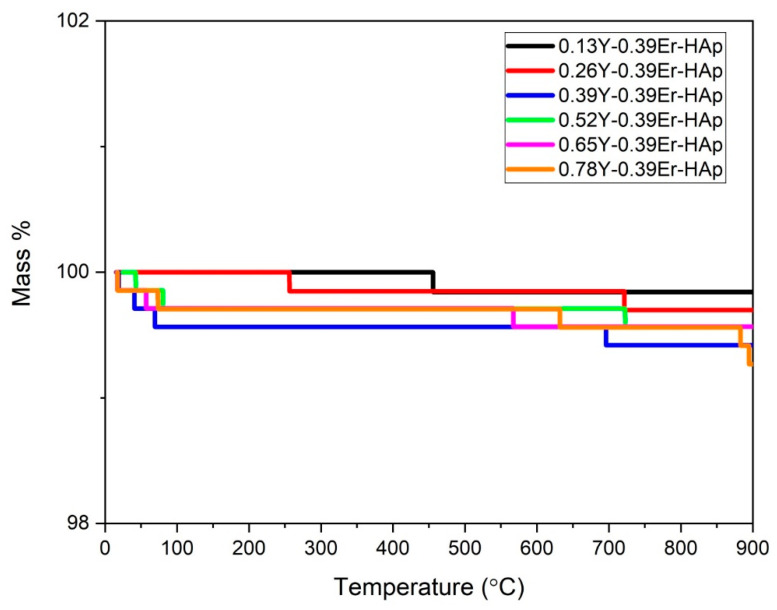
TGA thermograms.

Figure 6 and Figure 7 show DTA and TGA thermograms of as-prepared HAp samples co-doped with Er and Y. No peaks associated with the decomposition of the HAp structure in the DTA thermograms were found in Figure 6. Therefore, all of the samples were found thermally stable in the range of room temperature to 900 °C as per results reported by Ali et al. [43]. According to the study, Y concentration can improve and regulate the thermal stability of Er-doped HAps.

From room temperature to 900 °C, the TGA results in Figure 7 show no mass loss in the as-prepared samples. Moreover, 0.13Y-0.39Er-HAp, 0.26Y-0.39Er-HAp, 0.39Y-0.39Er-HAp, 0.52Y-0.39Er-HAp, 0.65Y-0.39Er-HAp, and 0.78Y-0.39Er-HAp exhibited total mass losses of 0.16, 0.30, 0.58, 0.43, 0.43, and 0.73%, respectively. These losses are related to the loss of adsorbed (between room temperature and 200 °C) and lattice water (between 200 °C and 400 °C) in the samples [44,45]. Dehydroxylation of the HAp samples results in the remaining mass losses for all samples at temperatures over 400 °C [3]. These mass losses were more clearly found at over 600 °C in the present investigation, and they are consistent with prior studies [46]. Figure 3 shows a decrease in the intensity of the band 3570 cm^−1^, which indicates dehydroxylation. Hence, the FTIR results support the mass losses due to the dihydroxylation process in the TGA results.

**Figure 8:**
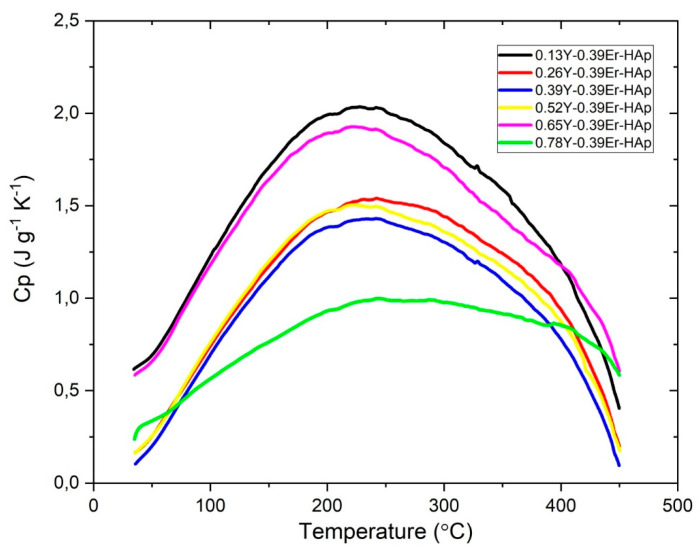
Heat capacity as a function of temperature plot for each sample.

The variation in specific heat capacity (Cp) dependent temperature is shown in Figure 8. One of the most prominent thermodynamic qualities of a substance is its specific heat capacity, whose value is affected by growing temperature and Y concentration, as illustrated in Figure 8. The entire Y-doped samples have much higher Cp values than the Er-HAp sample.

#### 3.1.5. In Vitro Biocompatibility Results of Y/Er-HAp Samples

**Figure 9:**
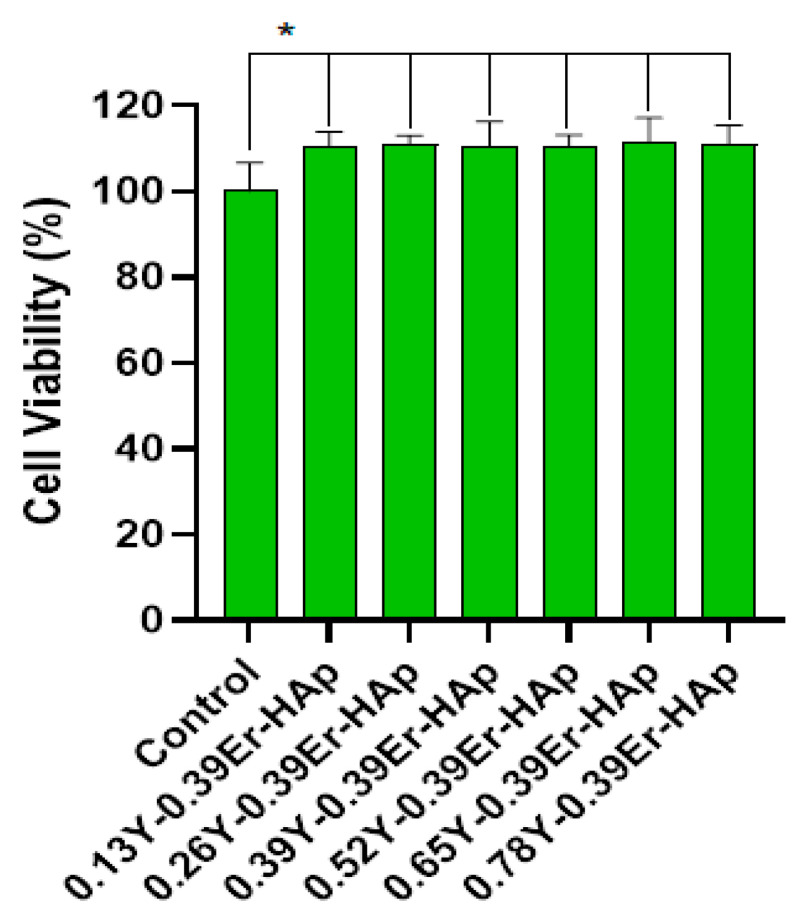
Cell viability level of L-929 cell exposed Y/Er-HAp samples. * Statistically significant (*p* < 0.05) in between control and treatment groups.

**Figure 10:**
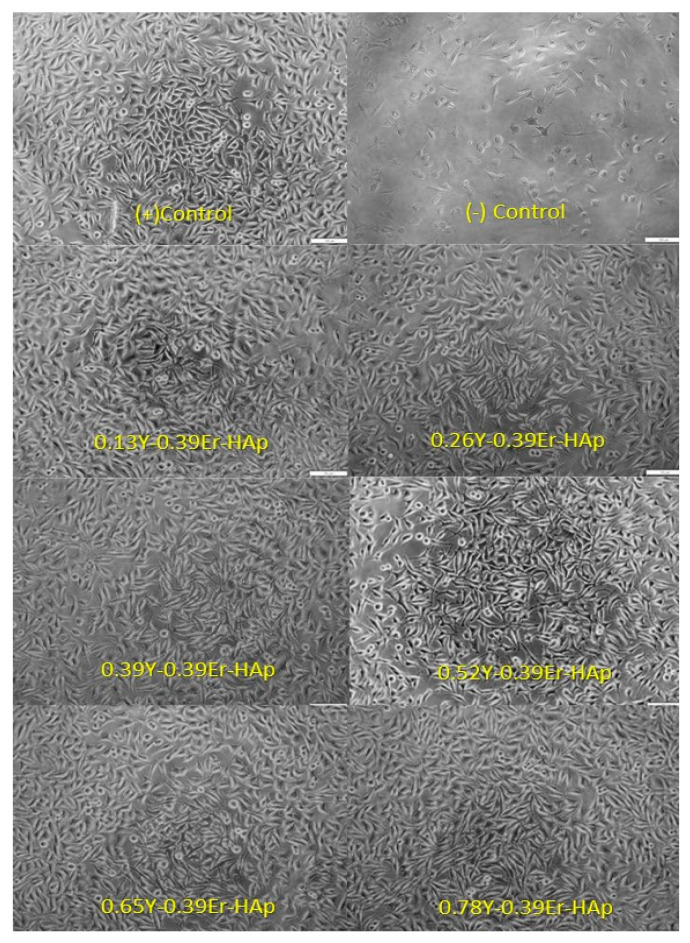
Optic microscope images for all the samples exposed to the L-929 cells.

In the biocompatibility test of the indirect method formulations, Mus musculus mouse fibroblast cells (L-929) were employed. L929 Mouse Fibroblast cells used in this biocompatibility study were commercially purchased from Thermo Fisher Scientific. Figure 9 and Figure 10 show the cell viability results and optical microscope images of the samples exposed to the L-929 cells, respectively. The cell viability percentages with their standard deviations are found to be 100.00 ± 6.62, 110.49 ± 3.34, 110.91 ± 2.10, 110.40 ± 5.90, 110.50 ± 2.65, 111.29 ± 5.89, and 110.97 ± 4.50 for the control, 0.13Y-0.39Er/HAp, 0.26Y-0.39Er/HAp, 0.39Y-0.39Er/HAp, 0.52Y-0.39Er/HAp, 0.65Y-0.39Er/HAp, and 0.78Y-0.39Er/HAp, respectively. Statistical analysis was performed for in vitro biocompatibility data. The differences were significantly determined between the control and treatment groups (*p* < 0.05). According to the findings, Y/Er-HAp samples had extremely high cell viability values, ranging between 110.10 and 111.29%. Because the samples looked to be more biocompatible than the control, they exhibited cell viability in more than 80% of all samples. As a result, we may conclude that all samples are biocompatible. The standard deviation in Figure 9 demonstrates how it varies based on the percentage of Y/Er-HAp samples. The microstructure of the doping elements may be the cause of the 0.39Y-0.39Er-HAp sample’s higher standard deviation than those of the other samples. When the percentage of Y changes, the deviation becomes less, at its lowest value for the 0.26Y-0.39Er-HAp. 

### 3.2. Theoretical Results

#### 3.2.1. Bandgap Structure and Density of States Calculations

The computed values of band structure and density of states of the materials were reported elsewhere [31]. This section sheds light on some of the reported data for the sake of completeness and overview. The electron density can determine as [47]:

(9) $$DOS\left(E\right)=\sum g\left(E-\varepsilon_{i}\right)$$

The number of states is multiplied by the likelihood that an electron will occupy each state in this calculation, which takes into consideration each energy state. Here *g*, *E,* and *ε_i_* are the Gaussian with a fixed FWHM, total energy, and energy of the *i*th molecular orbital, respectively.

Band structure and DOS measurements are based on the interaction of atoms inside a molecular structure. The interatomic distance between two or more atoms defines the number of splits in a solid molecule. Due to spin-orbit interactions and relativistic effects, the splitting of energy levels increases, which may change band structure and DOS in the structure [32].

**Figure 11:**
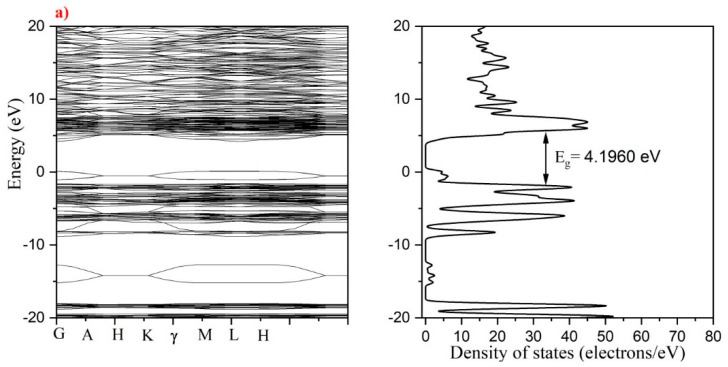

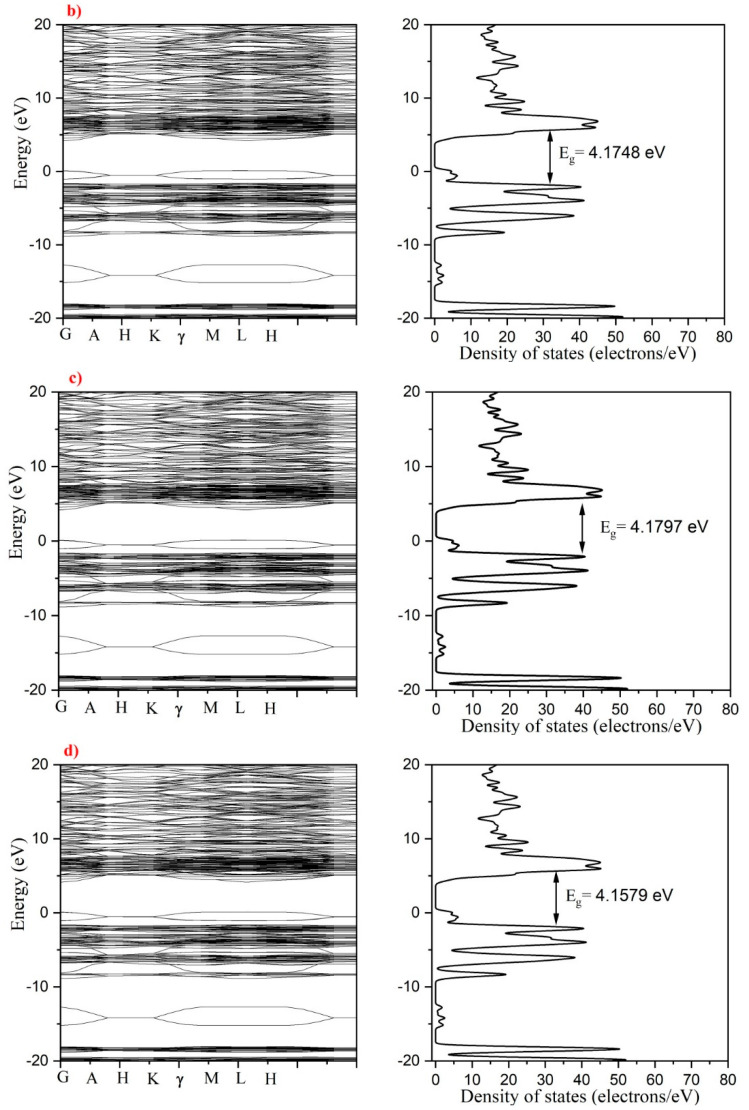

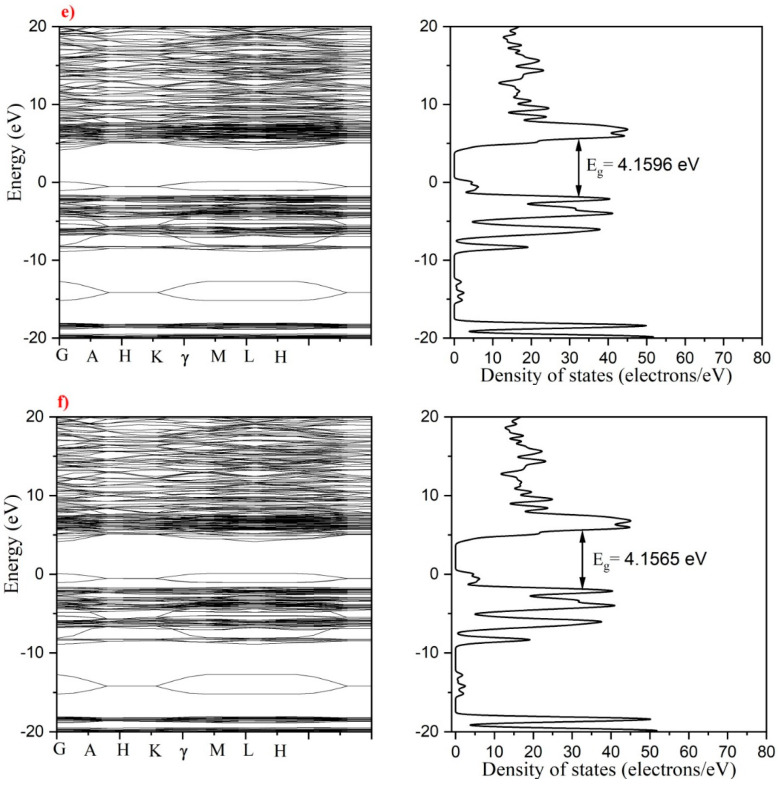
Band structure and density of states for (**a**) 0.13Y0.39Er/HAp, (**b**) 0.26Y0.39Er/HAp, (**c**) 0.39Y0.39Er/HAp, (**d**) 0.52Y0.39Er/HAp, (**e**) 0.65Y0.39Er/HAp, and, (**f**) 0.78Y0.39Er/HAp.

The computed DOS and band structure are shown in Figure 11a–f. For all calculations, the CASTEP program was utilized in conjunction with a DFT formulism [48]. The observed bandgap was measured at (G-H) intervals for all samples. In Figure 11a–f, the valence and conduction band surfaces appear to be virtually flat. The bandgap energy values for 0.13Y-0.39Er/HAp, 0.26Y-0.39Er/HAp, 0.39Y-0.39Er/HAp, 0.52Y-0.39Er/HAp, 0.65Y-0.39Er/HAp, and 0.78Y-0.39Er/HAp were found as 4.196 eV, 4.174 eV, 4.179 eV, 4.159 eV, 4.159 eV, and 4.156 eV, respectively. The electron configuration of Erbium (Er) is [Xe] 4f^12^6s^2^, whereas Yttrium has the form [Kr] 4d^1^5s^2^. The electrons of the Er atom other than 4f^12^6s^2^ are inner shell electrons in the Xenon configuration. The 4f level divides more than the 6s orbital because it is smaller and closer to limitation. The DOS related to 4f states exhibited contribution to the bands, whereas the s orbitals play a minor function in the electronic structure. By adding Y atoms in various concentrations, the energy bandgap of the molecule was somewhat decreased from 4.196 eV to 4.156 eV, as previously seen, except for 0.39Y-0.39Er/HAp, where the energy bandgap was increased. The reduction in bandgap energy may be related to changes in electronic states along symmetry lines, which could impact the bandgap value as a result of doping [32]. Due to its greater energy, this arrangement decreases the energy gap in the outcome when mixed with yttrium (Y) and other atoms in the HAp molecule.

#### 3.2.2. Density and Linear Absorption Coefficient Calculations

For 0.13Y-0.39Er-HAp, 0.26Y-0.39Er-HAp, 0.39Y-0.39Er-HAp, 0.52Y-0.39Er-HAp, 0.65Y-0.39Er-HAp, and 0.78Y-0.39Er-HAp, the density values were determined to be 3.1724, 3.1744, 3.1764, 3.1786, 3.1804, and 3.1824 g cm^−3^, respectively. The densities of Y (4.47 g cm^−3^) and Er (9.07 g cm^−3^) are greater than Ca (1.55 g cm^−3^) [49]. Because the concentration of Er was maintained constant, the addition of Y increased the density of the HAp system. As a result, the larger the concentration of Y causes, the higher the density.

**Figure 12:**
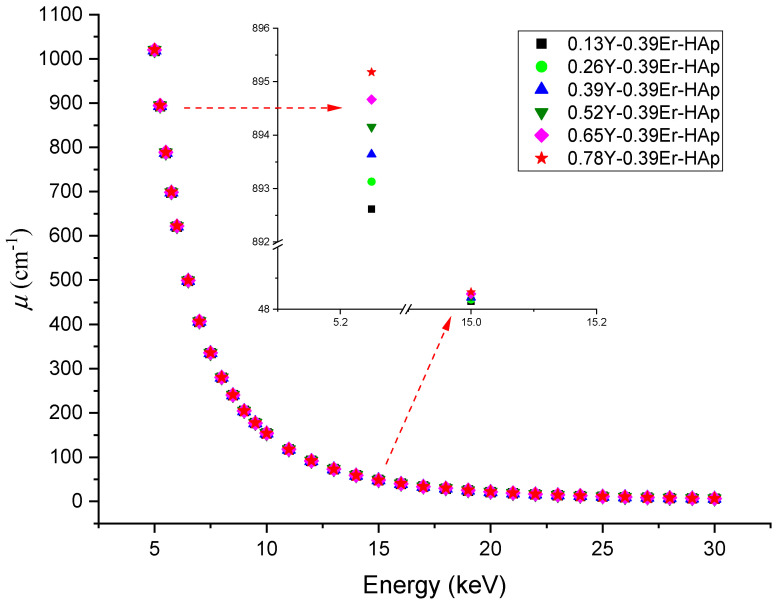
Energy-dependency of the linear absorption coefficient for all the samples.

Figure 12 shows the linear absorption coefficient (LAC) as a function of photon energy. As photon energy increases, all structures notice a decrease. Furthermore, when the amount of Y present increases, the LAC value increases. Due to their electromagnetic radiation attenuation properties, all samples are ideal candidates for radiation shielding and medical applications, and the observed results are highly compatible with the literature [32,47,50,51].

## 4. Conclusions

In the present study, Er-based HAps doped with Y in various concentrations (e.g., 0.13, 0.26, 0.39, 0.52, 0.65, and 0.78 at. %) were synthesized using the wet chemical technique. It was found that the Y dopant affected the lattice parameters. The crystallite size and crystallinity percentage were observed to vary considerably. When the Y element was inserted into Er-based HAp, lattice strain and stress were produced. Raman and FTIR spectroscopy confirmed the HAp structure within all the investigated samples. The Y concentration has a substantial impact on the thermal behavior of the Er-Based HAp. The bandgap energy of the samples decreased dramatically from 4.196 eV to 4.156 eV when the Y concentration increased, according to the theoretical results. None of the samples showed any signs of cytotoxicity. Cell viability was lower than the control group except for the sample 0.39Y-0.39Er/HAp. The linear absorption coefficient declines as the Y concentrations increase. The entire series of samples appeared biocompatible and can be employed in biomedical applications. 

## Figures and Tables

**Table 1:** The amount of each chemical used in the synthesis.

Sample	Formulation	Ca(NO_3_)_2_·4H_2_O (mmol)	Er(NO_3_)_3_·5H_2_O (mmol)	Y(NO_3_)_3_·6H_2_O (mmol)	(NH_4_)_2_HPO_4_ (mmol)
0.13Y-0.39Er-HAp	Ca_9.948_Y_0.013_Er_0.039_(PO_4_)_6_(OH)_2_	49.740	0.195	0.065	30.000
0.26Y-0.39Er-HAp	Ca_9.935_Y_0.026_Er_0.039_(PO_4_)_6_(OH)_2_	49.675		0.130	
0.39Y-0.39Er-HAp	Ca_9.922_Y_0.039_Er_0.039_(PO_4_)_6_(OH)_2_	49.610		0.195	
0.52Y-0.39Er-HAp	Ca_9.909_Y_0.052_Er_0.039_(PO_4_)_6_(OH)_2_	49.545		0.260	
0.65Y-0.39Er-HAp	Ca_9.896_Y_0.065_Er_0.039_(PO_4_)_6_(OH)_2_	49.480		0.325	
0.78Y-0.39Er-HAp	Ca_9.883_Y_0.078_Er_0.039_(PO_4_)_6_(OH)_2_	49.415		0.390	

**Table 2:** The comparison of theoretical and experimental values of the lattice parameter and unit cell volume.

	*Theoretical*			*Experimental*		
Samples	*a* (nm)	*c* (nm)	*V* (nm)^3^	*a* (nm)	*c* (nm)	*V* (nm)^3^
0.13Y-0.39Er-HAp	0.93269	0.67899	0.51151	0.94069	0.68215	0.52275
0.26Y-0.39Er-HAp	0.93165	0.67970	0.51091	0.94029	0.68632	0.52549
0.39Y-0.39Er-HAp	0.93208	0.67932	0.51109	0.94131	0.68234	0.52358
0.52Y-0.39Er-HAp	0.93261	0.67893	0.51138	0.93852	0.68569	0.52304
0.65Y-0.39Er-HAp	0.93108	0.68013	0.51060	0.93727	0.68499	0.52111
0.78Y-0.39Er-HAp	0.93166	0.67947	0.51074	0.93663	0.68414	0.51976

**Table 3:** The XRD-related calculated parameters.

Samples	*X_C_%*	*D_S_* (nm)	*D_WH_* (nm)	*ε* × 10^−4^	*σ* (MPa)	*u* (kJ m^−3^)
0.13Y-0.39Er-HAp	87.40	35.55	25.58	−11.500	−84.653	542.219
0.26Y-0.39Er-HAp	83.66	37.20	42.40	3.904	35.514	57.124
0.39Y-0.39Er-HAp	86.40	31.71	24.98	−8.790	120.487	9.391
0.52Y-0.39Er-HAp	87.29	36.67	35.01	−1.370	35.238	1.162
0.65Y-0.39Er-HAp	86.90	32.01	24.32	−10.500	−40.061	247.890
0.78Y-0.39Er-HAp	84.34	29.25	19.78	−17.500	114.546	66.597

**Table 4:** Theoretical and experimental values of the Ca/P and (Ca+Er+Y)/P ratios.

Sample	Ca/P (Theo.)	Ca/P (Exp.)	(Ca+Er+Y)/P (Theo.)	(Ca+Er+Y)/P (Exp.)
0.13Y-0.39Er-HAp	1.6580	1.6517	1.6667	1.6654
0.26Y-0.39Er-HAp	1.6558	1.6547	1.6667	1.6682
0.39Y-0.39Er-HAp	1.6537	1.6510	1.6667	1.6716
0.52Y-0.39Er-HAp	1.6515	1.6408	1.6667	1.6607
0.65Y-0.39Er-HAp	1.6493	1.6450	1.6667	1.6673
0.78Y-0.39Er-HAp	1.6472	1.6331	1.6667	1.6535
